# Three Amino Acid Residues Bind Corn Odorants to McinOBP1 in the Polyembryonic Endoparasitoid of *Macrocentrus cingulum* Brischke

**DOI:** 10.1371/journal.pone.0093501

**Published:** 2014-04-04

**Authors:** Tofael Ahmed, Tian-tao Zhang, Zhen-ying Wang, Kang-lai He, Shu-xiong Bai

**Affiliations:** 1 State Key Laboratory for the Biology of the Plant Diseases and Insect Pests, Institute of Plant Protection, Chinese Academy of Agricultural Sciences, Beijing, China; 2 Bangladesh Sugarcane Research Institute, Ishurdi, Pabna, Bangladesh; Russian Academy of Sciences, Institute for Biological Instrumentation, Russian Federation

## Abstract

Odorant binding proteins (OBPs) play a central role in transporting odorant molecules from the sensillum lymph to olfactory receptors to initiate behavioral responses. In this study, the OBP of *Macrocentrus cingulum* McinOBP1 was expressed in *Escherichia coli* and purified by Ni ion affinity chromatography. Real-time PCR experiments indicate that the McinOBP1 is expressed mainly in adult antennae, with expression levels differing by sex. Ligand-binding experiments using N-phenyl-naphthylamine (1-NPN) as a fluorescent probe demonstrated that the McinOBP1 can bind green-leaf volatiles, including aldehydes and terpenoids, but also can bind aliphatic alcohols with good affinity, in the order trans-2-nonenal>cis-3-hexen-1-ol>trans-caryophelle, suggesting a role of McinOBP1 in general odorant chemoreception. We chose those three odorants for further homology modeling and ligand docking based on their binding affinity. The Val58, Leu62 and Glu130 are the key amino acids in the binding pockets that bind with these three odorants. The three mutants, Val58, Leu62 and Glu130, where the valine, leucine and glutamic residues were replaced by alanine, proline and alanine, respectively; showed reduced affinity to these odorants. This information suggests, Val58, Leu62 and Glu130 are involved in the binding of these compounds, possibly through the specific recognition of ligands that forms hydrogen bonds with the ligands functional groups.

## Introduction

Olfaction is a finely tuned sense, essential for sensory assessment of the environment, which plays a crucial role for insects in foraging, host-seeking, mating, ovipositing and avoiding toxin substances. Olfaction is mediated by specific olfactory sensory neurons, which project their dendrites into a lymphatic cavity where odorant binding proteins (OBPs) are present at high concentrations [1]. The major proteins involved in the selectivity and sensitivity of the insect olfactory system are odorant-binding proteins (OBPs) [2] and odorant receptors (ORs) [3]. The small, water soluble polypeptides OBPs are present at the interface between the external environment and chemoreceptors [4]. The first step in the recognition of odorants are air-born small hydrophobic molecules mediated by OBPs [5], [6] that are involved in odorant reception, where they bind, solubilize and deliver odorant molecules to ORs [2], [7]. ORs are heterodimers comprised of highly variable odorant-binding subunits associated with one conserved co-receptor (OR83b) [8] and these are most widely expressed in the dendritic membranes of olfactory sensory neurons (OSNs) that are housed in sensory hairs called olfactory sensilla [9].

The insect OBP was discovered at the beginning of 1980s in the giant moth *Antheraea polyphemus* [10]. Many studies related to function and structure have occurred, since their discovery [10], [11], [12], [13], yet mode of action and specific role in chemoreception of OBPs remains largely unknown [4]. In the last few years, several studies have demonstrated requirement of OBPs for chemoreception in insects, which has lead to useful models. Studies demonstrate electrophysiological responses are abolished when *A. polyphemus* OBPs are removed [14]. *Acyrthosiphon pisum* OBP3 was shown to interact with its alarm pheromone, (E)-β-farnesol [15]. Recently studies show presence of OBPs increases sensitivity and specificity to the olfactory system of *Bombyx mori* [16], [17]. In *Drosophila melanogaster* OBP76 LUSH gene knock out, abolishes response to 11-*cis*-vaccenyl acetate (cVA), but is reactivated when the gene is expressed [18]. So, OBPs are not only involved in odorant detection, but also the discrimination of olfaction stimuli in certain insect behaviors [4]. The specific behavioral response is linked to two genes that encode OBPs, namely 57d and 57e that are found in *D. simulans* and *D. sechellia* [19]. The specific OBP was deficient in each of 17 strains of *D. melanogaster*, which influenced fly responses to several odorants [20].

Interactions between insect OBPs and ligand-binding have been explored by considering key amino acid residues, 3D (dimensional) structures and their physiochemical properties [4], [21], [22]. Studies show that the pheromone-binding protein 1 (BmorPBP1) of *B. mori* binds to bombykol when Ser56 interacts with hydroxyl group in the binding pocket [23]. The C-terminal segment of BmorPBP1 is unstructured at pH 7.0, but a major conformational change occurs at pH<7.0, when the seventh α-helix enters into the binding pocket [24]. The acidic pH of the membrane surface triggers the conformational change [4]. Several studies report that this type of conformational change is clearly observed with several OBPs [4], [25]. Other OBPs, due to the short length of their C-terminus, cannot make an additional helix and push into the binding cavity, but covers the binding pocket with a lid, possible to contribute to the binding of specific ligands [26], [27].

*Drosophila* LUSH seems to have another mechanism. The single amino acid residue is involved with the conformational change when this protein binds with cVA. LUSH undergoes a conformational change that triggers binding of the complex to the specific olfactory receptor [4], which generates the electrophysiological response of olfactory receptor OR67d in the T1 neuron [28]. This has been very elegantly demonstrated by showing a mutant of LUSH, which mimicks the conformation of the protein when bound to cVA, activates the electrophysiological signal from the corresponding neuron, even in the absence of the pheromone [28].

The M*acrocentrus cingulum* Brischke (Hymenoptera: Braconidae) is distributed widely in Europe, Japan, Korea and China [29] and is considered as a specialist larval polyembryonic endoparasitoid of the genus *Ostrinia* (Lepidoptera: Crambidae) [30], [31]. Parasitic effectiveness of *M. cingulum* depends on location and infection by pathogens and other factors like temperature and humidity. Female *M. cingulum* oviposit in all instars but prefer second to fourth [32]. Parasitization rates are variable, but typically high, for example, reaching up to 80% in studies conducted in Northern France [33], [34]. In our present research, we have identified a novel OBP protein gene, McinOBP1 (GenBank accession no. KF900276) from the antennal cDNA library of *M. cingulum*; which was expressed and purified *in vitro*. We also have measured the fluorescence binding affinities of McinOBP1 to 23 corn odorants belonging to three groups: aldehydes, terpenoids, and aliphatic alcohols with others aromatic compounds. On the basis of binding affinity results, we predicted the putative binding sites of McinOBP1 by three dimensional structure modeling and molecular docking methods. We confirm these predictions with site-directed mutagenesis experiments and fluorescence binding assays. The expression patterns of McinOBP1 in different tissues of male and female adults also were detected with real-time quantitative PCR (qPCR).

## Materials and Methods

### Insects

*M. cingulum* were collected from *O. furnacalis* larvae living on corn plants at the Langfang Experiment Station of the Institute of Plant Protection, Chinese Academy of Agricultural Sciences, China. A laboratory colony was established on host larvae of *O. furnacalis* that were reared on an artificial diet as described by Zhou et al. [35] and maintained at 25°C with a photoperiod of 16 h:8 h, L:D. Adult parasitoid wasps were fed with 20% honey solution.

### RNA Extraction and cDNA Synthesis

Antennae, heads with maxillary palps (excluding antennae), legs, thoraxes and abdomen of female and male individuals were dissected 1–2 days after eclosion and immediately frozen in liquid nitrogen, then stored at –80°C until RNA extraction. Total RNA was extracted from the antennae or other tissues using TRIzol reagent (Invitrogen, Carlsbad, CA, USA), following the manufacturer’s instructions. Before transcription, the RNAs were treated with DNase I (Invitrogen) to remove residual genomic DNA. cDNA was prepared from total RNA by reverse transcription, using the RT-PCR system (Promega) in accordance with the manufacturers protocol.

### Polymerase Chain Reaction (PCR)

Aliquots of 1 μL of crude cDNA were amplified in a Bio-Rad Gene CyclerTM thermocycler with gene specific primer as follows: McinOBP1F: 5′-CCCATGGCAGAAAACGCGGATCCT-3′; McinOBP1R: 5′-CGAAGCTTTTAGTTGCCTGGAGCTCGG-3′.

The restriction enzymes are *NcoI* and *HindIII* in the forward and reverse primer, are underlined, respectively. The PCR condition consisted of an initial 3 min step at 94°C followed by 37 cycles of 94°C for 30 sec, 56°C for 30 sec and 72°C for 1 min and a final 10 minute step at 72°C.

### Expression Pattern of McinOBP1

Real-time quantitative PCR (qPCR) was conducted to detect the relative expression levels of McinOBP1 in adult male and female different tissues of *M. cingulum*. The RNA/cDNA preparation of each tissue was performed in triplicate. The gene specific primer was designed using Primer express 5.0 as follows: McinOBP1-F: 5′-CAACTCAATCGGTGGCAGAAG-3′; McinOBP1-R: 5′-TTGCATAGATCGTCGACAGGTT-3′. The reference gene, β-actin (accession number EU585777) was used in qRT-PCR equipment. The gene specific primer and β-actin were used to measure the C_t_ values of the cDNA templates to ensure the C_t_ values were between 22 and 25.

Real-time qPCR experiments were performed using 96 well plates (Applied Biosystems, Carlsbad, CA), ABI Prism 7500 Fast Detection System (Applied Biosystems, Carlsbad, CA) and Brilliant II SYBR Green qPCR master mix (Takara). qRT-PCR was conducted in 20 μL reactions containing 50× SYBR Premix Ex Taq 10 μL, primer (10 mM) 0.4×2 μL, ROX reference dye II 0.4 μL (50×), sample cDNA 1 μL, sterilized ultra-pure grade H_2_O 7.8 μL. Cycling conditions were: 95°C for 30 sec, 40 cycles of 95°C for 05 sec and 60°C for 30 sec. Afterwards, the PCR products were heated to 95°C for 15 sec, cooled to 60°C for 1 min and heated to 95°C for 15 sec to measure the dissociation curves. No-template and no-reverse transcriptase controls were included in each experiment. To check reproducibility, each test sample was done in triplicate technical replicates and three biological replicates. Relative quantification was performed by using the comparative 2^−ΔΔCT^ method [36]. All data were normalized to endogenous β-actin levels from the same tissue samples and the relative fold change in different tissues was calculated with the transcript level of the abdomen as calibrator. Thus, the relative fold change in different tissues was assessed by comparing the expression level of each OBP in other tissues to that in the abdomen.

For the verified the ΔCT values, each sample was diluted 5 serial fold (1, 10, 100, 1000, 10000). The amplifications were performed from each dilution in triplicate by using the gene specific primer and β-actin. The absolute values of the slopes of all lines from the template dilution plots (log cDNA dilution vs. ΔCT) were close to zero (data not shown). Quantification of gene transcript level, in the ΔΔCT calculation for the comparative 2^−ΔΔCT^ method assumes that the amplification efficiencies of the target and reference are approximately equal.

### Cloning and Sequencing

The PCR products were ligated into the pGEM-T Easy vector (Promega, Madison, WI, USA), using a 1∶5 (plasmid:insert) molar ratio and incubated overnight at 4°C. The ligation products were transformed into TransT1 *E. coli* competent cell and grown on LB solid medium with 10 mg/mL ampicillin, 1 M isopropyl β-D-thiogalactoside (IPTG) 40 μL, and X-gal 40 μL. Positive colonies were selected by PCR using the plasmid’s primers SP6 and T7, grown in LB liquid medium with ampicillin and sequencing.

### Cloning of McinOBP1-Wild Type in Expression Vectors

The pGEM plasmid containing the positive clones and the pET32a (+) were digested with *NcoI* and *HindIII* restriction enzymes for 2 hrs at 37°C and then the products were separated on agarose gel. The target fragments were purified from the gel and ligated into the digested pET32a (+) plasmid and the recombinant plasmids were transformed into TransT1 competence cells and grown on LB solid medium with 10 μl ampicillin (10 mg/mL). Selected colonies were grown in LB liquid medium with ampicillin and then incubated in a shaker at 200 rpm, *E. coli* and transformed into BL21 (DE3)-competent cells. A single clone was identified and cultivated overnight in LB liquid medium including ampicillin on a shaker at 200 rpm in 37°C. The resulting plasmids were sequenced and shown to encode the mature proteins.

### Purification of Proteins

pET32a (+) vectors containing the verified sequences encoding the McinOBP1-wt and mutants proteins were grown overnight in 5 mL LB with 10 mg/mL ampicillin. The cultures were diluted to 1∶100 in fresh medium with ampicillin and then shaken at 200 rpm for 2.5 hrs at 37°C. Then, 0.5 mM IPTG was added to the culture and incubated for additional 5 hrs for the induction of target proteins. The bacterial cells were collected by centrifugation and re-suspended in 1× phosphate-buffered saline (PBS), then sonicated; the pellet and supernatant were collected by centrifuge and analyzed by 12% SDS-PAGE. Proteins were present both in supernatant and inclusion body. We chose the supernatant expressed proteins for further uses. Proteins were purified by means of Ni ion affinity chromatography then desalted and concentrated. The purified proteins were digested with rProtease to remove the His-tag. The size and purity of proteins were checked by 12% SDS-PAGE analysis. The concentrations of the proteins were measured along with Bradford method [37] by the microplate reader Multiskan Ascent (Thermo Labsystem, Germany).

### Preparation of Site-Directed Mutants

The three mutants of McinOBP1, m1 (mutation of amino acid, valine to alanine at 58 position), m2 (mutation of amino acid, leucine to proline at 62 position) and m3 (mutation of amino acid, glutamic to alanine at 130 position), were developed using the kit of Quick-change lightning site-directed mutagenesis (Stratagene, La Jolla, CA, USA). The McinOBP1-wt/pGEM-T Easy construct was used as a template. The primers were designed manually and mutation sites are underlined as listed below.

Val58: GTC to GCC for McinOBP1-m1 mutant.

Forward primer: 5′-GCTACGCCTCTTGTTTTC-3′.

Reverse primer: 5′-GAAAACAAGAGGCGTAGC-3′.

Leu62: CTG to CCG for McinOBP1-m2 mutant.

Forward primer: 5′-CTCTTGTTTTCCGCAAAACATCG-3′.

Reverse primer: 5′-CGATGTTTTGCGGAAAACAAGAG-3′.

Glu130: GAG to GCG for McinOBP1-m3 mutant.

Forward primer: 5′-GTTATATGCGATGCTGGG-3′.

Reverse primer: 5′- CCCAGCATCGCATATAAC-3′.

The PCR conditions were 95°C for 3 min for initial denaturation, followed by 37 cycles of 95°C for 30 sec, 57°C for 1 min and final extension at 72°C for 10 min. The selected mutants were subcloned into pGEM-T Easy vector (Promega) then sequenced. The same expression vector and competent cells were used as for McinOBP1-wt. The mutant’s expression and purification were performed as described for the wild-type protein.

### Fluorescence Binding Assays

Fluorescence binding assays were recorded on Shimadzu fluorescence spectrophotometer (Shimadzu, Japan) with a 1 cm light path quartz cuvette; both excitation and emission silts widths were 5 nm. The probe was excited at 337 nm and emission spectra were recorded between 350 and 500 nm. To measure the affinity of the fluorescence ligand N-phenyl-1-naphthylamine (1-NPN) to McinOBP1-wt and mutants, a 2 μM solution of the protein in 50 mM Tris-HCl, pH 7.4, was titrated with aliquots of 1 mM ligand in high-performance liquid chromatography (HPLC) purity grade methanol to final concentrations of 2–20 μM while the other competitor were measured in competitive binding assays, where 1-NPN using as the fluorescent reporter at 2 μM concentration and each competitor (odorants) over concentration ranges 2–12 μM. For determining the bound ligand from the intensity values of fluorescence emission assuming that the proteins were 100% active, with a stoichiometry of 1∶1 (protein:ligand) at saturation. The GraphPad Prism 5 (GraphPad Software, Inc.) was used to estimate the K_1–NPN_ (K_D_ of protein complex/1-NPN) values by nonlinear regression for a unique site of binding. Scatchard plots were used to linearize the curves. For other competitor, IC_50_ (concentrations of competitor halving the initial fluorescence value of 1-NPN) value was calculated using Microsoft Office Excel 2010, and the dissociation constants (K_D,_ which correspond to the concentration of ligand at which the binding site on a particular protein is half occupied) were calculated according to *K_D_* = [IC_50_]/[1+(1–NPN)/*K*_1-NPN_], where (1-NPN) is the free concentration of 1-NPN and *K*_1-NPN_ is the dissociation constant of protein complex/1-NPN [4], [38], [39].

### Molecular Modeling and Ligands Docking

A three dimensional model of McinOBP1 was generated using the I-TASSER Protein Structure and Function Predictions web server (http://zhanglab.ccmb.med.umich. edu/I-TASSER/) [40], [41]. The sequence of McinOBP1 was compared to all known protein sequences that had a high sequence similarity with the target sequence on RCSB Protein Data Bank (PDB) (www.rcsb.org). The homology modeling of McinOBP1 was performed using build homology model of AaegOBP1 [27]. The best model was confirmed using the evaluation of PDF total energy, verify score and Ramachardran plot.

Molecular docking can fit molecules together in a favorable configuration to form a complex system. Three dimensional chemical structures were used to search for trans-2-nonenal, cis-3-hexen-1-ol and trans-caryophelle on PubChem Compound (www.pubchem.ncbi.nlm.hih.gov), where the structural information from the theoretically modeled complex may helped clarify the binding mechanism between McinOBP1 and odorants. The advanced docking program CDOCKER [42] was used to perform the automated molecular docking to gain insights into binding mode of McinOBP1 with these three odorants. CHARMm-based molecular dynamics scheme was used to dock ligands into a receptor binding site in the CDOCKER program. The energy minimization was used to refine the ligand poses. The ligand-protein docked complexes were selected according to their interaction energy with geometric matching quality. Interaction energy was calculated from odorants and key residues of McinOBP1.

## Results

### Coding and Sequence Analysis of McinOBP1

**Figure 1:**
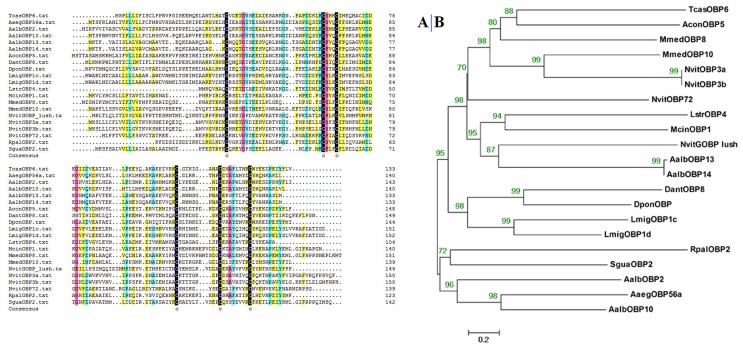
Alignments and Phylogenetic analysis of McinOBP1. (A) Alignment of McinOBP1(KF900276) from hymenopteran insects as well as other insects. *Microplitis mediator* (MmedOBP8, AEF14409)\ *Rhynchophorus palmarum* (RpalOBP2, AAD31883)\ *Tribolium castaneum* (TcasOBP5, EFA05677)\ *Aedes aegypti* (AaegOBP56a, XP_001658489)\ *Nasonia vitripennis* (NvitGOBP lush, XP_001603472)\ Aedes albopictus (AalbOBP10, AGI04311)\ *Nasonia vitripennis* (NvitOBP3a, CCD17772)\ *Nasonia vitripennis* (NvitOBP3b, CCD17860)\ *Sclerodermus guani* (SguaOBP2, ABE68831)\ *Microplitis mediator* (MmedOBP10, AEO27860)\ *Dendroctonus ponderosae* (DponOBP, ENN77432)\ *Aedes albopictus* (AalbOBP14, GI04314)\ *Aedes albopictus* (AalbOBP2, AFC60563)\ *Aedes albopictus* (AalbOBP13, AGI04313)\ *Delia antiqua* (DantOBP8, BAI82448)\ *Argyresthia conjugella* (AconOBP5, AFD34173)\ *Locusta migratoria* (LmigOBP1e, ACR39385)\ *Laodelphax striatella* (LstrOBP4, AEQ19910)\ *Nasonia vitripennis* (NvitOBP72, CCD17841). Six conserved cysteine residues are highlighted with black background (B) Phylogenetic tree of McinOBP1 amino acid sequences in others insects.

McinOBP1of the *M. cingulum* was obtained from the antennal cDNA library is a protein specifically expressed in antennae. By using gene specific primers, a full length cDNA encoding McinOBP1 was cloned. Sequence analysis showed that the full length (ORF) consists of 423 nucleotides that encode 140 amino acids residues, with a predicted MW of 15.71 kD. For McinOBP1 SingnalP predicted a peptide with 20 amino acid residues, with calculated molecular weight and isoelectric point of 13.46 kDa and 4.73, respectively. An alignment of the amino acid sequences of McinOBP1 with the corresponding OBP from other species of Hymenoptera and additional insects is shown in Figure 1A. McinOBP1 had the typical six-cysteine signature of OBPs with a common pattern: C_1_–X_15–39_–C_2_–X_3_–C_3_–X_21–44_–C_4_–X_7–12_–C_5_–X_8_–C_6_, (X is any amino acid). The phylogenetic tree was constructed based on the amino acid sequences of OBP from other insects (Fig. 1B). The dendrogram shows that the McinOBP1 has closer ancestry with insects from the same order. This suggests McinOBP1 is a classical OBP.

### Tissues-Specificity Expression Pattern of McinOBP1

**Figure 2:**
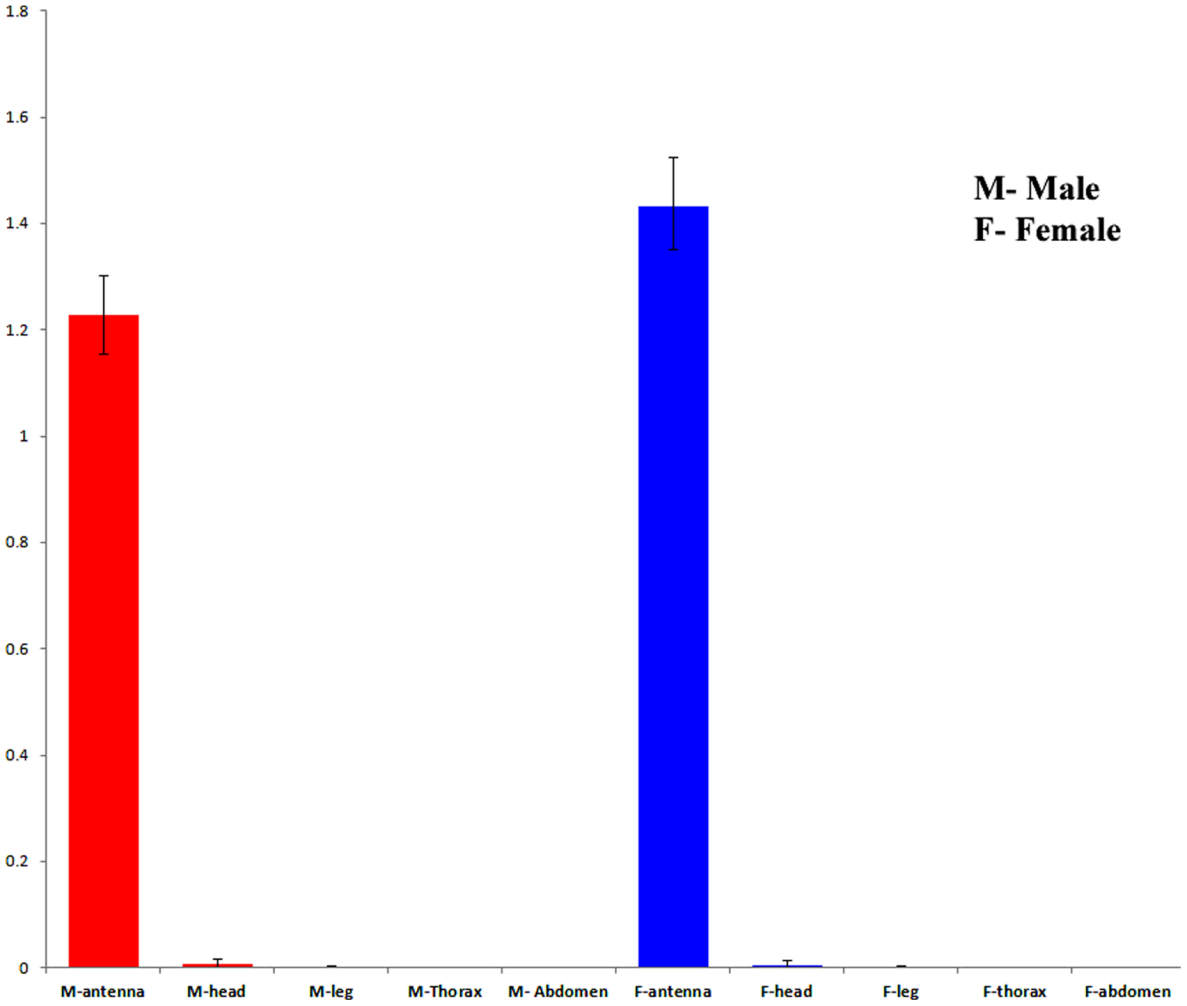
Expression pattern analysis of McinOBP1. Transcript level of *M. cingulum* odorant binding protein 1 (McinOBP1) in different tissues of adult male and females measured by qPCR. cDNAs were amplified with specific primers from antennae, heads (without antennae),thoraxes, abdomens, legs, and abdomen.

We examined the expression pattern of McinOBP1 in different tissues (male and female antennae, de-antennated heads, legs, thoraxes, and abdomens) by qPCR. Total RNA of each sample was isolated and separated from male and female that was revers-transcribed; the expected product was largely amplified in male and female antennae, with low transcripts in other tissues, suggesting that McinOBP1 is mainly expressed in adult antennae, with expression levels differing by sex (Fig. 2). In general, the levels of transcripts were very low in all tissues except the antennae, where McinOBP1 was highly expressed.

### Recombinant Protein McinOBP1-wt Expression and Purification

**Figure 3:**
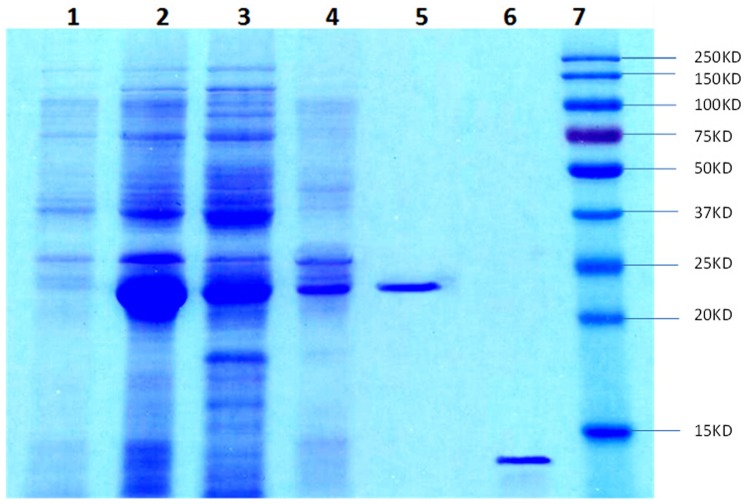
SDS-PAGE analysis of expressed recombine McinOBP1. Lane-1: Non-induced pET32a (+)/McinOBP1 transformed BL21 (DE3) cells; 2: IPTG induced *E. coli* pET32a (+)/McinOBP1 transformed BL21(DE3) cells; 3. Supernatant after ultrasonic treated pET32a (+)/McinOBP1 cells; 4: Inclusion body of pET32a (+)/McinOBP1 cells; 5: Ni–NTA columns purified proteins; 6. Purified protein cleaved His-tag by rProtease; 7: Molecular weight marker.

The recombinant McinOBP1-wt (wild type) protein was expressed in *E. coli* as a soluble protein in supernatant with high yield (∼10 to 20 mg/L). The recombinant protein was directly purified from the total protein (supernatant) by the Ni ion affinity chromatography. The His-tag was digested from the purified recombinant protein by rProtease, and then Ni ion affinity chromatography was used to separate His-tagged and uncleaved His-tagged protein (Fig. 3). The purified recombinant protein, McinOBP1-wt was then used for the further experiments in this study.

### Ligand-Protein Binding Specificity

**Figure 4:**
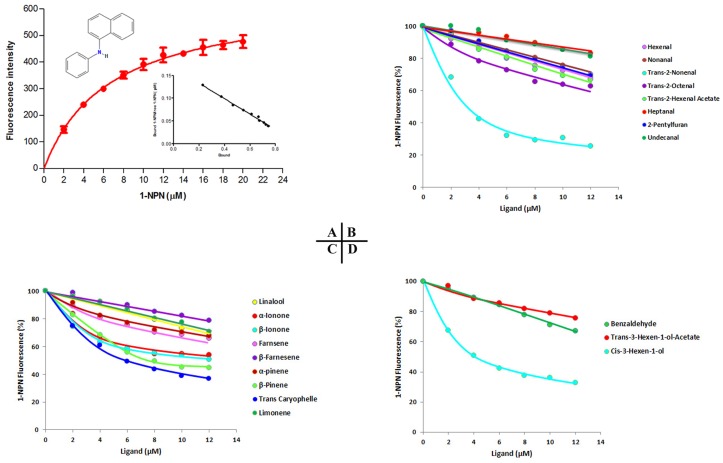
Binding of 1-NPN and various ligands to McinOBP1. (A) Binding curves and relative Scatchard plot (insert) of N-phenyl-1-naphthylamine (1-NPN) and McinOBP1 protein. Two μM solutions of protein was titrated with 1 μM solution of 1-NPN in methanol to final concentrations of 2–20 μM. (B) Competitive binding affinities of McinOBP1-wt to different aldehyde ligands. (C) Binding affinities of various terpenoids to McinOBP1-wt and (D) Affinities of McinOBP1-wt to aliphatic alcohol and others.

The binding affinity of the fluorescence probe 1-NPN (N-phenyl-1-naphthylamine) to protein was measured with the excitation wavelength at 337 nm and emission spectra were recorded between 350 and 500 nm. The dissociation constant of 1-NPN-bound recombinant McinOBP1-wt, approximately 6.60 μM, was calculated according to the changes in fluorescence intensity (Fig. 4A), which was used to calculate the dissociation constants (K_D_) of ligands.

The competitive binding affinities of McinOBP1-wt to 23 ligands belonging to aldehyde, terpenoids and aliphatic alcohol with others aromatic compounds are listed in Table 1; and curves for a few representative ligands tested are shown in Figure 4B–4D. Table 1 lists the IC_50_ values (concentration of ligand that reduces by half the initial fluorescence value), and when possible, the calculated dissociation constants (K_D_) for OBP/ligand combination.

**Table 1:** IC_50_ values and calculated dissociation constants, **K_D_** (μM) for the complexes between McinOBP1-wt and different ligands at pH = 7.4.

Ligands	IC_50_	K_D_	CAS Number
**Aldehydes**			
Hexenal	18.92	16.43	66-25-1
Nonanal	19.61	17.03	124-19-6
Trans-2-Nonenal	3.40	2.96	18829-56-6
Trans -2-Hexenal Acetate	11.92	10.35	6728-26-3
Trans-2-Octenal	15.69	13.63	2548-87-0
Heptanal	35.54	30.86	111-71-7
2-Pentylfuran	19.13	16.61	3777-69-3
Undecanal	–	–	112-44-7
Decanal	–	–	112-31-2
2,6 dimethyl octane	–	–	2051-30-1
**Terpenoids**			
Linalool	18.62	16.17	78-70-6
α-Ionone	12.12	14.30	127-41-3
β-Ionone	10.33	8.97	14901-07-6
Farnsene	10.28	8.93	502-61-4
β-Farnesene	28.49	24.75	18794-84-8
α-pinene	17.83	15.48	80-56-8
β-Pinene	12.67	10.79	19902-08-0
β- Caryophelle	7.51	6.53	87-44-5
Limonene	21.05	18.28	138-86-3
**Aliphatic alcohols and others**			
Benzaldehyde	17.92	15.56	100-52-7
Cis-3-Hexen-1-ol	4.61	4.00	3681-71-8
Trans-3-Hexen-1-ol-Acetate	23.85	20.72	928-97-2

Ligands concentrations that exceeded 50 μM for half-maximal inhibition are represented as ‘−’ and were not used for calculating Dissociation constants *K*i are marked as ‘−’.

IC_50_, ligand concentration displacing 50% of the fluorescence intensity of the McinOBP1/N-phenyl-1-naphthylamine complex; CAS, Chemical Abstracts Service;; *K*_D_, dissociation constant.

**Figure 5:**
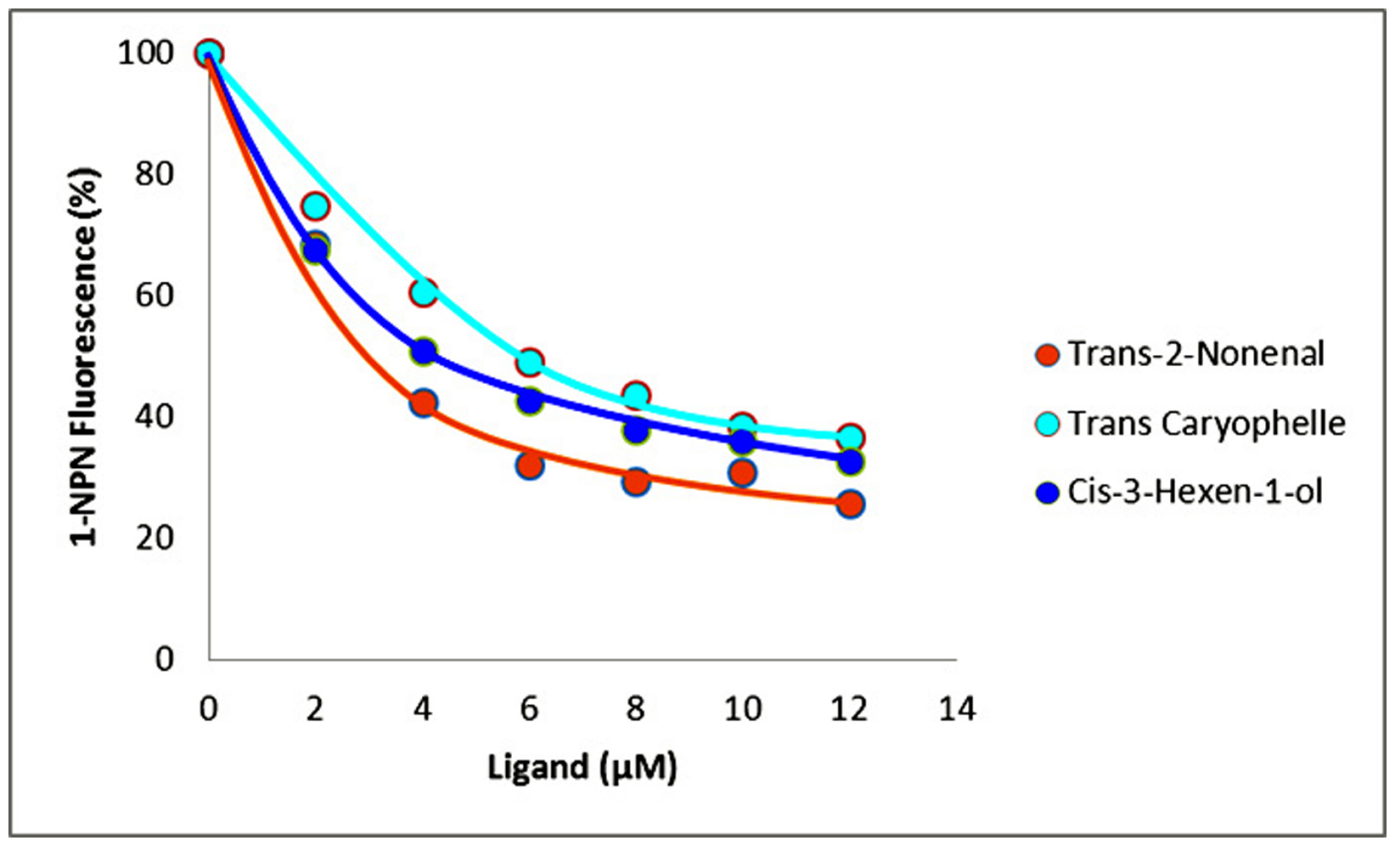
Three ligands binding curves. Affinities of selected odorants: trans-2-nonenal, cis-3-hexen-1-ol, and β-caryophelle to *M. cingulum* odorant-binding protein 1 (McinOBP1). The fluorescence intensities are plotted against increasing ligand concentrations.

The binding affinities results indicate that most of the tested ligands succeeded in displacing 1-NPN from the McinOBP1/1-NPN complex. Amongst the odorants, trans-2-nonenal, cis-3-hexen-1-ol and trans-caryophelle had the highest binding affinities to McinOBP-wt with K_D_ values of 2.96±1.18, 4.00±1.44 and 6.52±2.03 μM, respectively (Fig. 5). Decanal, undecanal and 2, 6 dimethyl octane did not bind to McinOBP1-wt. The α-ionone, β-ionone, farnesene, β-pinene, and cis-3-hexenyl acetate had medium binding abilities to McinOBP1-wt, with dissociation constants of 9.30±0.38, 8.97±0.63, 8.93±0.29, 10.79±0.51, and 10.35±0.71 μM, respectively.

### Molecular Modeling of McinOBP1-wt Protein and Ligands Docking

**Figure 6:**
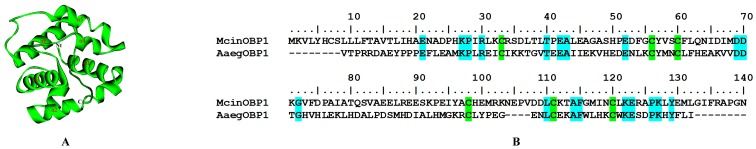
3D structure model of the *M. cingulum* odorant binding protein 1. (A) Predicted 3D model of McinOBP1 was built on the reference of structure of OBP1 from the Mosquito *Aedes aegypti*. Six *α*-helixes, N-terminal (Nt) and C-terminal (Ct) are marked. (B) Sequence alignment of McinOBP1 and AaegOBP1. In the alignment of the two proteins, conserved cysteines are highlighted in green and identical residues are highlighted in light blue.

Sequence of McinOBP1-wt was compared to all known proteins with AaegOBP1 as template in order to model the 3D structure of McinOBP1-wt with I-TASSER. Following the homology modeling, the best model (Fig. 6) was chosen from 10 candidates, and its quality was further checked by Ramachardran plot and verification score (Data not shown). C-score for McinOBP1 of 0.24 (ranges −5 to 2) suggests high confidence in the model [41]. Estimated TM-score and RMSD of predicted model McinOBP1-wt, are 0.68±0.12 and 5.1±3.3Å, respectively.

**Figure 7:**
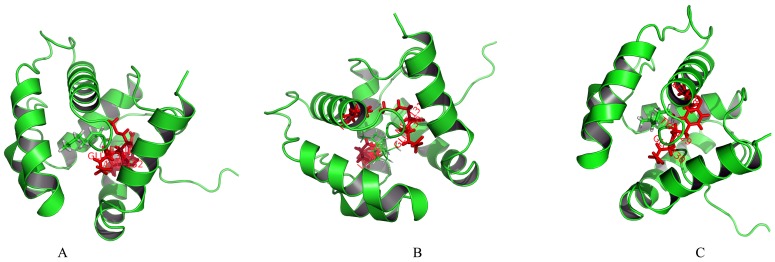
Docking of *M. cingulum* odorant binding protein 1 with ligands. Molecular docking of McinOBP1-wt with (A) trans-2-nonenal; (B) cis-3-hexen-1-ol and (C) β-caryophelle. The predicted key amino acids residues are shown in red and respective ligands shown in green.

Considering the three dimensional model of McinOBP1, most of the residues were confirmed to have hydrophobicity at their binding site. Ligand poses and consensus score programs were used to evaluate binding pose affinities for the residues. Subsequently, the optimal 3D binding conformations of protein ligand complexes were selected Figures 7A-7C. The interaction energies between key residues and the ligands are listed in Table 2. All of the residues are located in the cavity formed by six helices. From the docking simulation results, several residues including Phe54, Val58, Phe61, Leu62, IleE65, Ile67, Phe74, Ala79, Val83, Leu87, Ser91, Ile95, Leu121, Leu128 and Glu130 seemingly play important roles in the binding of McinOBP1-wt to trans-2-nonenal, cis-3-hexen-1-ol and trans-caryophelle.

**Table 2:** The interaction energies (kcal/mol) between the key residues of McinOBP1-wt and ligands.

Residues	Trans-2-Nonenal			Cis-3-Hexen-1-ol			Trans-Caryophelle		
	*E*_total_	*E*_vdw_	*E*_ele_	*E*_total_	*E*_vdw_	*E*_ele_	*E*_total_	*E*_vdw_	*E*_ele_
Phe54	−0.763	−0.392	−0.371	−	−	−	−	−	−
Val58	−1.625	−1.445	−0.180	−2.797	−2.351	−0.446	−2.361	−2.244	−0.117
Phe61	−1.098	−1.019	−0.078	–	–	–	−1.370	−1.351	−0.019
Leu62	−2.137	−1.369	−0.768	−2.201	−1.039	−1.161	−2.709	−2.724	0.015
Ile65	–	–	–	−0.318	−0.300	−0.017	−1.203	−1.263	0.060
Ile67	–	–	–	−0.868	−0.855	−0.013	−1.411	−1.427	0.015
Phe74	−0.953	−1.133	0.179	–	–	–	−0.828	−0.848	0.020
Ala79	−0.412	−0.395	−0.016	−0.652	−0.615	−0.037	−1.123	−1.191	0.067
Val83	−0.387	−0.391	0.004	–	–	–	−0.430	−0.447	0.017
Leu87	−0.220	−0.292	0.072	–	–	–	−0.193	−0.217	0.023
Ser91	−0.964	−1.097	0.133	–	–	–	−0.463	−0.503	0.039
Ile95	−0.746	−0.793	0.046	−0.264	−0.259	−0.005	−0.895	−0.946	0.050
Leu121	−1.182	−1.277	0.095	−1.718	−1.829	0.110	−1.187	−1.115	−0.072
Leu128	–	–	–	−1.087	−0.940	−0.146	−1.435	−1.426	−0.009
Glu130	−5.548	−2.226	−3.321	−2.846	−1.239	−1.607	−3.475	−2.937	−0.537

*E*_total_, total interaction energy; *E*_vdw_, Van der Waals energy; *E*_ele_, electrostatic interaction energy.

### Mutagenesis and Binding Assays

**Figure 8:**
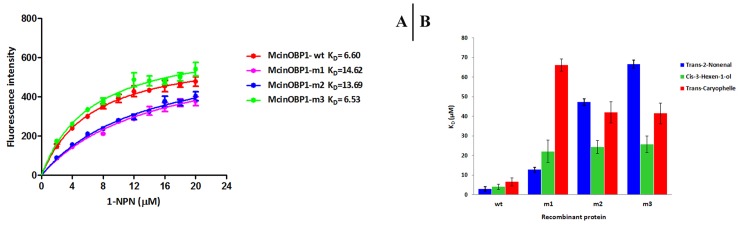
Binding of 1-NPN and ligands to McinOBP1 mutants. (A) Affinities of 1-NPN to the McinOBP1-wt and three mutants. Dissociation constants (K_D_) were 6.60 μM for McinOBP1-wt, 14.62 μM for McinOBP1-m1, 13.69 μM for McinOBP1-m2 and 6.53 μM for McinOBP1-m3 are shown in inset. (B) Dissociation constants of complexes between McinOBP1-wt and its three mutants (McinOBP1-m1to 3) and the three selected odorants. These three mutants showed lower binding affinity to selected three odorants with respect to the wild type, indicating that the respective amino acids are involved in binding these ligands.

On the basis of molecular modeling and ligand docking simulation results, Val58, and Glu130 were mutated to alanine, and Leu62 was mutated to proline. These recombinant mutant proteins were expressed and purified as described for the wild type protein and analyzed by SDS-PAGE. The same fluorescence probe 1-NPN was used under the previously described conditions. The mutants Val58, Leu62 and Glu130 bind 1-NPN dissociation constants of 14.62±1.07, 13.69±0.71, and 6.53±0.50 μM, respectively, (Fig. 8A). This was calculated from the ligand dissociation constants (K_D_) shown in Table 3.

**Table 3:** Affinities of McinOBP1-wt and three mutants to trans-2-nonenal, cis-3-hexen-1-ol and trans- caryophelle.

Proteins	Trans-2-Nonenal		Cis-3-Hexen-1-ol		Trans-Caryophelle	
	IC_50_ (μM)	K_D_ (μM)	IC_50_ (μM)	K_D_ (μM)	IC_50_ (μM)	K_D_ (μM)
McinOBP1-wt	3.40	2.96	4.61	4.00	7.51	6.53
McinOBP1-m1	13.56	12.69	23.58	22.07	70.69	66.16
McinOBP1-m2	50.61	47.17	26.07	24.29	45.01	41.95
McinOBP1-m3	76.73	66.54	29.48	25.57	47.75	41.41

Competitive binding affinities of mutants to trans-2-nonenal, cis-3-hexen-1-ol and trans-caryophelle were measured and are shown in Figure 8B. Results show that all three mutant proteins have lower binding affinities to trans-2-nonenal, cis-3-hexen-1-ol and trans-caryophelle compared to the McinOBP1-wt protein. Binding affinity of mutant m1 (Val58) to trans-caryophelle was decreased considerably (K_D_ = 66.16±3.04 μM). Similarly, mutant m3 (Glu130) binding affinity to trans-2-nonenal decreased (K_D_ = 66.55±2.01 μM). Mutants m2 (Leu62) and m3 (Glu130) also had lower binding affinities to trans-caryophelle (K_D_ = 41.95±5.46 and 41.42±5.38 μM, respectively); and mutants m1, m2 and m3 had lower binding affinities to Cis-3-hexen-1-ol (K_D_ = 22.07±5.72, 24.30±3.31 and 25.57±4.25 μM, respectively).

## Discussion

Insects recognize complex environmental stimuli such as volatile and non-volatile compounds with their well-developed olfactory system. This system plays an important role in several behaviors, such as searching for hosts, food, mates, evading predators and locating oviposition sites. Insect OBPs are very diverse proteins with an average of only 14% amino acid identity [43]. OBPs are soluble polypeptides present at the interface between the external environment and chemoreceptors for insects and other animal species [4]. McinOBP1 gene was obtained from our previous study about the construction of antennal cDNA library of *M. cingulum* (unpublished). Alignment analysis of McinOBP1 shows it has 6 typical conservative cysteines in the sequence (Fig. 1A), which is consistent with previous reports [44]. Quantitative examination of transcript levels shows McinOBP1 is expressed in male and female antennae but there is very low if any expression in others tissues (Fig. 2). These results are similar to those found in other insects [45], [46], [47], [48]. Some of these studies suggested that McinOBP1 plays an important role in the olfactory systems response to chemical stimuli.

In this study to better understand McinOBP1 structure, function and binding characteristics, site directed mutagenesis and a fluorescence binding specificity were conducted. Odorants analogues of chemicals released from damaged corn plants of Asian corn borer (ACB), *Ostrinia furnacalis* (Guenée) [49] were selected as ligands. A total 23 odorants were tested, including ten aldehydes, nine terpenoids and four aliphatic alcohols/aromatic compounds. Three odorant compounds (decanal, undecanal and 2,6 dimethyl octane) have no binding affinity to McinOBP1. Among the tested compounds, trans-2-nonenal, trans-2-octenal, trans-2-hexenal acetate, trans-caryophelle, β-pinene, β-ionone, and cis-3-hexen-1-ol have binding affinities to McinOBP1. In general, “β” isomer odorants have higher binding affinities than “α” isomer, which suggests McinOBP1 could discriminate the chiral structure of chemical molecules. Future research, however, needs to investigate whether odorant solubility affects binding capacity. Furthermore, a limitation to note is fluorescence assays only provide an indirect measure of binding affinity [50], [51].

From the binding assay results, McinOBP1not only binds green-leaf volatiles, including aldehydes and terpenoids, but also binds aliphatic alcohols, in the order trans-2-nonenal>cis-3-hexen-1-ol>trans-caryophelle (Fig. 5). The total interaction energy of trans-2-nonenal with Val58 and Leu62 were −1.625 and −2.137 kcal/mol in the val58A/trans-2-nonenal and Leu62/trans-2-nonenal complex, respectively; these are higher than Val58/Leu62/cis-3-hexen-1-ol (−2.797 and −2.201 kcal/mol) and Val58/Leu62/trans-caryophelle complex (−2.361 and −2.709 kcal/mol), which may be the reason for strong binding affinity to trans-2-nonenal than cis-3-hexen-1-ol and trans-caryophelle (Table 2). The Glu130/cis-3-hexen-1-ol complex showed strong binding affinity with the interaction energy of −2.846 kcal/mol followed by −3.475 and −5.548 kcal/mol in the Glu130/trans-caryophelle and Glu130/trans-2-nonenal complex, respectively (Table 2). This suggests McinOBP1 act as a general OBP that binds to general odorants of insect food sources. Volatile compounds are emitted from undamaged, mechanically injured or herbivore damaged plants. Parasitoids used plant volatiles (HIPVs) and green-leaf volatiles (GLVs) as chemical cues for locate hosts [52], [53]. In this study, trans-2-nonenal, cis-3-hexen-1-ol and trans-caryophelle had the highest binding affinities to McinOBP1-wt. These compounds are known attractants to ACB [49], the meadow moth, *Loxostege sticticalis* L. [54] and the braconidae wasp *Microplitis mediator* Haliday [55]. So, McinOBP1 could play an important role in the chemoreception and host location of *M. cingulum*.

Our modeling suggests trans-2-nonenal, cis-3-hexen-1-ol and trans-caryophelle bind to McinOBP1 in a cisoid conformation similar to bombykol in BmorGOBP2 [21]. Formation of hydrogen bonds of hydrophilic amino acid residues are involved in the recognition of ligands [50]. Hydrogen bonds make networks to hold the ligand in the central cavity through the locked C-terminus. Similar results were reported with PBP-ligand interactions in *Lymantria dispar* [56]. In the moth *Helicoverpa armigera,* HarmOBP7 binds with good affinity to both pheromone components Z-11-hexadecenal and Z-9-hexadecenal as well as linear aldehydes, alcohols and esters.

Binding affinity of insect OBP is influenced by pH. A study reported that binding affinities of HarmOBP7 and a mutant lacking the last 6 residues do not substantially decrease in acidic conditions, but increases at basic pH values, with no significant differences between wild-type and mutant [4]. Furthermore, a second mutant, where C-terminus one (Lys123) of the three lysine residues was replaced by methionine showed reduced affinity to pheromone components, as well as to their analogues due to less hydrogen bonds formation affinity [4]. pH-dependent ligand-release mechanisms were reported in *Culex quinquefasciatus*, CquiOBP1 [57], *Aedes aegypti*, AaegOBP1 [27] and *Anopheles gambiae*, AgamOBP1 [58], in which a decrease in pH, disrupts the hydrogen bond between the C-terminal loop and the rest of the protein, and then opens the loop to release the ligands [50].

We measured binding affinities of trans-2-nonenal, cis-3-hexen-1-ol and trans-caryophelle to the mutant McinOBP1-m1 and m2 (Fig. 8B). Unlike 1-NPN, whose binding to selected odorants was not affected by the change of the respective amino acids, mutant McinOBP1 showed poorer affinities compared to the wild type McinOBP1. The trans-caryophelle showed the weakest binding affinity with McinOBP1-m1 whereas trans-2-nonenal had the lowest binding affinity with McinOBP1-m2 (Fig. 8B). The third mutant McinOBP1-m3 binds 1-NPN with a dissociation constant slightly better (K_D_ = 6.53±0.50 μM) than the wild type, indicating that the mutation did not significantly affect binding and that Glu130 is not involved in binding 1-NPN (Fig. 8A). However, binding of these three odorants to McinOBP1-m3 was much weaker than to the wild type (Fig. 8A). These results suggest Glu130 is involved in binding to aldehyde odorants, as well as alcohol and terpenoid odorants. The molecular modeling of three mutants, McinOBP1m1-m3 did not change the overall confirmation of the protein structure, McinOBP1-wt (supplementary Fig. S1). These results also confirm the observation, reported with other OBPs [59], [60], that different ligands bind equally well to the OBP. Presumably, different orientations in the binding pocket make it possible to for, different residues to bind.

These results suggest McinOBP1 is a general OBP that plays an important role in recognizing several plant volatiles. The three amino acids Val58, Leu62 and Glu130 appear to be involved in binding the aldehyde trans-2-nonenal as aldehyde and the terpenoid trans-caryophelle. Seemingly these compounds interact with McinOBP1 with their hydrophobic chain inside the binding pocket and the functional group on the mount of the cavity. Future investigations with NMR tests or X-ray diffraction are needed to confirm these ligand-OBP interaction findings.

## Supporting Information

Figure S1 **3D model structure of McinOBP1-wt, McinOBP1-m1, McinOBP1-m2 and mcinOBP1-m3.** (A) Predicted 3D protein structure of McinOBP1-wt, (B) 3D protein structure of McinOBP1-m1, (C) 3D protein structure of McinOBP1-m2, and (D) 3D protein structure of McinOBP1-m3. Six *α*-helixes, N-terminal (Nt) and C-terminal (Ct) are marked.
(TIF)
