# Supplementary figures and images for: Three Amino Acid Residues Bind Corn Odorants to McinOBP1 in the Polyembryonic Endoparasitoid of Macrocentrus cingulum Brischke
Source: PLoS One. 2014 Apr 4;9(4):e93501. doi: 10.1371/journal.pone.0093501 (PMC3976273; doi:10.1371/journal.pone.0093501)

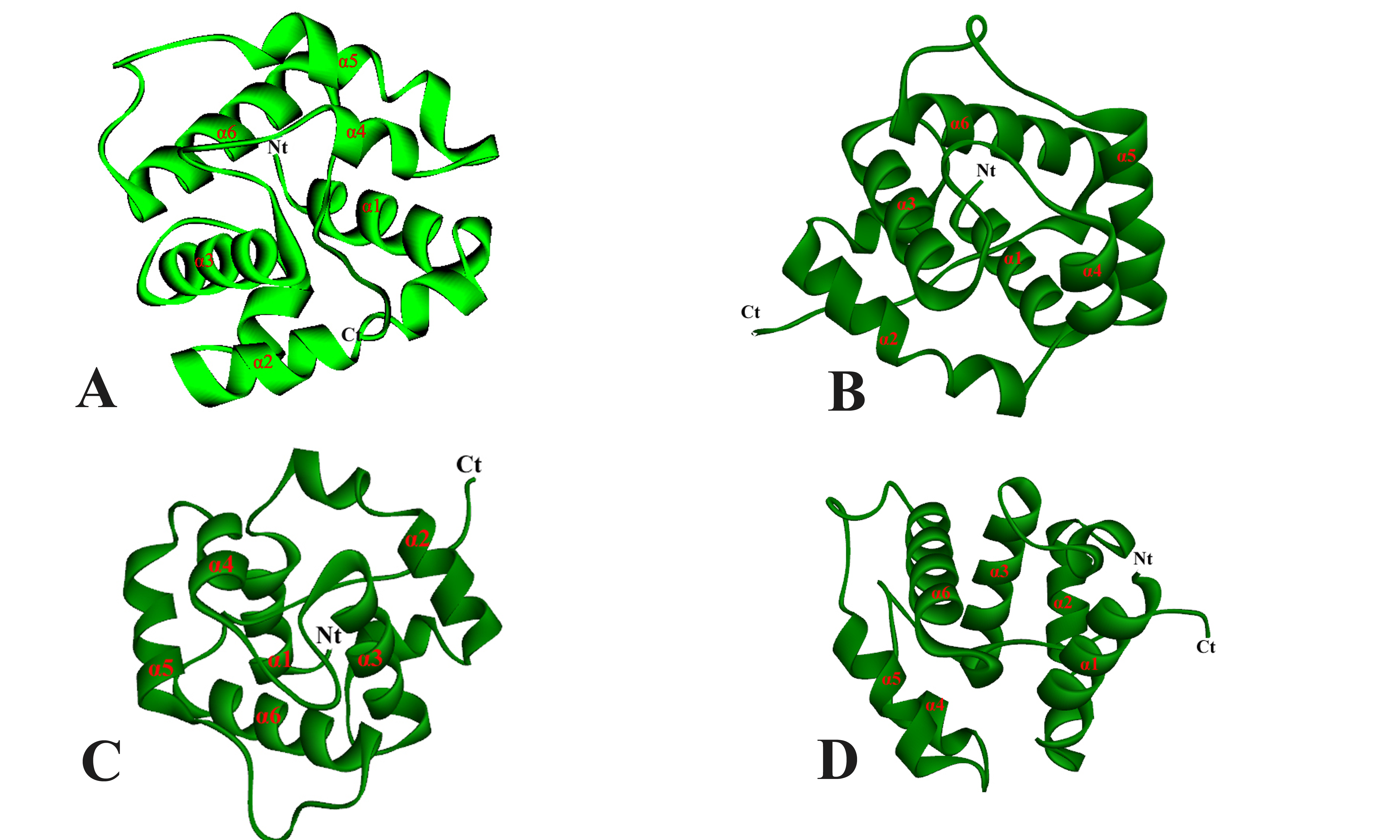

Supplement: Figure S1 — 3D model structure of McinOBP1-wt, McinOBP1-m1, McinOBP1-m2 and mcinOBP1-m3. (A) Predicted 3D protein structure of McinOBP1-wt, (B) 3D protein structure of McinOBP1-m1, (C) 3D protein structure of McinOBP1-m2, and (D) 3D protein structure of McinOBP1-m3. Six α-helixes, N-terminal (Nt) and C-terminal (Ct) are marked. (TIF) [file pone.0093501.s001.tif]
